# Limb Amputations in Fixed Dystonia: A Form of Body Integrity Identity Disorder?

**DOI:** 10.1002/mds.23671

**Published:** 2011-04-11

**Authors:** Mark J Edwards, Araceli Alonso-Canovas, Arnette Schrag, Bastiaan R Bloem, Philip D Thompson, Kailash Bhatia

**Affiliations:** 1 Sobell Department of Motor Neuroscience and Movement Disorders, Institute of NeurologyUCL, London, United Kingdom; 2 Department of Clinical Neurosciences, Institute of Neurology, UCL, Royal Free CampusLondon, United Kingdom; 3 Department of Neurology, Donders Institute for Brain, Cognition and Behavior, Radboud University Nijmegen Medical CentreNijmegen, The Netherlands; 4 Department of Neurology and University Department of Medicine, Royal Adelaide Hospital and University of AdelaideAdelaide, South Australia

**Keywords:** fixed dystonia, amputation, psychogenic, chronic regional pain syndrome

## Abstract

Fixed dystonia is a disabling disorder mainly affecting young women who develop fixed abnormal limb postures and pain after apparently minor peripheral injury. There is continued debate regarding its pathophysiology and management. We report 5 cases of fixed dystonia in patients who sought amputation of the affected limb. We place these cases in the context of previous reports of patients with healthy limbs and patients with chronic regional pain syndrome who have sought amputation. Our cases, combined with recent data regarding disorders of mental rotation in patients with fixed dystonia, as well as previous data regarding body integrity identity disorder and amputations sought by patients with chronic regional pain syndrome, raise the possibility that patients with fixed dystonia might have a deficit in body schema that predisposes them to developing fixed dystonia and drives some to seek amputation. The outcome of amputation in fixed dystonia is invariably unfavorable. © 2011 Movement Disorder Society

Traditional medical dualism polarizes opinion as to whether fixed dystonia is best characterized as a psychogenic or an organic disorder. In typical cases, young women develop a fixed abnormal posture of a limb following minor peripheral injury, associated with severe pain in the absence of any identifiable ongoing nerve or tissue injury.^1^ Contractures, abnormal nail growth, muscle wasting, and skin color and temperature change can occur, indicating the maintenance of postures and limb immobilization even when unobserved.^1^ There are some similarities,^2^–^4^ but also important differences^5^ on electrophysiological tests between fixed and primary (mobile) dystonia. Many (but perhaps not all) patients with fixed dystonia fulfill criteria for psychogenic dystonia and/or somatization disorder.^1^

We recently described an abnormality of mental rotation in patients with fixed dystonia.^6^ In this test patients with fixed dystonia were slower at performing a mental rotation of a body part displayed in different orientations on a screen than were healthy controls. The ability to perform mental rotation of corporeal objects is linked to the concept of body schema,^7^ and therefore these data support the hypothesis that patients with fixed dystonia may have a distortion of body image.^6^ Similar abnormalities in mental rotation have been reported in primary dystonia^8^ but may be modulated in a different fashion in fixed dystonia (see below). Body image disturbances have been described in various psychiatric disorders including eating disorders, schizophrenia, depression, depersonalization syndrome, and dysmorphophobia.^9^ There is also a hierarchy of neurological syndromes of disturbed body awareness, ranging from neglect, asomatognosia, and misoplegia to limb personification (where the affected limb is thought to belong to someone else) in diseases of the parietal lobe. Body image disturbances also are part of the syndromes of phantom limbs, alien limbs, and anarchic hands.^9^

Further evidence of a body image disturbance in fixed dystonia is presented in 5 patients with fixed dystonia who sought (and in most cases achieved) amputation of the affected limb. These cases were not collected systematically, but are drawn from our combined clinical experience of approximately 200 cases of fixed dystonia. We draw parallels with the phenomenon of seeking amputation in fixed dystonia and “apotemnophilia,” or the compulsion to amputate healthy limbs, that is considered part of the spectrum of body integrity identity disorders.^10^, ^11^ On the basis of our cases and previous data with regard to amputation in complex regional pain syndrome type 1 (CRPS1), we speculate that fixed dystonia is itself part of the spectrum of body integrity identity disorders.

## Case Summaries

### Case 1

A 36-year-old woman suffered a superficial left foot injury at age 21 with contralateral spread and a diagnosis of CRPS1. She was originally classified as having probable psychogenic dystonia. Subsequently, an above-knee amputation of the left leg was performed. This was followed by severe phantom limb and back pain. A spinal stimulator was inserted at another hospital without much benefit. The patient remained hospitalized for 4 years. Prolonged immobility led to sacral pressure sores. She refused to eat at will and rejected nursing care, resulting in malnutrition and weight loss that necessitated PEG tube feeding. At present, 14 years after onset, she remains hospitalized. A psychiatric diagnosis of severe personality disorder was made.

### Case 2

A 27-year-old woman with osteoporosis was originally classified as having probable psychogenic dystonia after developing fixed dystonia of the left foot at age 21 following a peripheral injury with contralateral spread. She fulfilled diagnostic criteria for CRPS1. Four years after onset, she had an above-knee amputation of the affected leg and subsequently developed phantom limb pain. She has an indwelling suprapubic catheter for unexplained urinary retention and underwent a loop ileostomy for unexplained constipation/fecal incontinence 1 year later.

### Case 3

A 34-year-old man 12 years previously developed inward turning of the left foot after falling down stairs and injuring the left foot at work. The inturning of the foot initially progressed for a few years and spread to the other side, but improved when the partner he was very dependent on left him. He remained stable over the following years, but the foot was painful on passive movement and he had painful back spasms. He was wheelchair bound. He had additional episodes of body tremor and developed nonepileptic seizures. A diagnosis of somatoform disorder was made, with urinary, gastrointestinal, and skin symptoms and trance states, depression, anxiety disorder, and an eating disorder. He was categorized as having a clinically established psychogenic movement disorder. He had tried multiple pain and spasticity medications, botulinum toxin, and bilateral tibial nerve blocks (with transient remission for 1 year) and requested an amputation of the affected limb.

### Case 4

A 46-year-old woman with bipolar affective disorder and epilepsy had developed a right-sided fixed wrist and finger flexion at age 25 following an accident to her right hand. The symptoms increased following right wrist surgery and spread to the other hand following a minor injury to the left hand. These were associated with CRPS1 and psychogenic signs, and she was diagnosed with a probable psychogenic movement disorder. Botulinum toxin injections led to initial improvement in the painful spasms without functional improvement, but later deterioration occurred. A sympathectomy did not provide benefit, and she requested an amputation of the affected limbs.

### Case 5

**FIG. 1:**
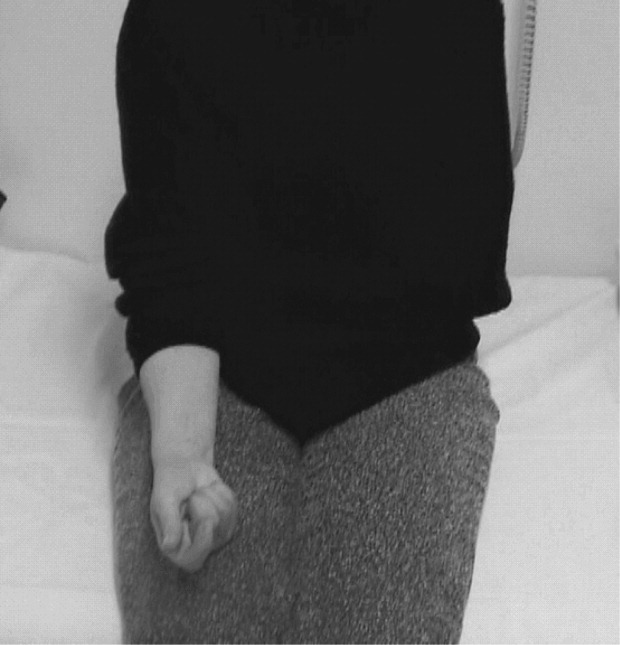
Case 5 showing amputation on the left with fixed dystonic posturing on the right, which developed after amputation of the left arm.

This 48-year-old woman (see Fig. 1) had sustained a left-sided wrist fracture 8 years earlier. In the following year, she noticed progressive dystonic posturing of the left hand, with a fixed clenching of the third, fourth, and fifth digits, with relative sparing of the index finger and thumb. Treatment for dystonia (including oral baclofen, anticholinergics, and benzodiazepines, as well as local botulinum toxin injections) was ineffective. She also experienced progressive and incapacitating pain in the left arm, which eventually led to amputation 3 years after the initial wrist fracture. In the year following the left arm amputation, she developed a very similar clinical presentation in the right arm. She also developed progressive pain in the stump of the left arm, similar to the previous pain she had experienced in the dystonic hand and forearm. She is now seeking amputation of the right arm.

## Discussion

A review of the medical literature on patients who seek amputation reveals 2 broad groups. In the first, patients with an apparently normally functioning limb without physical signs or pain seek amputation of the limb (apotemnophilia). In the other, patients experience chronic limb pain and seek amputation to remove the painful limb.

### People Without Physical Signs Who Request Amputation—Apotemnophilia or Body Integrity Identity Disorder

The concept of body integrity identity disorder (BIID) is derived from case reports in the 1970s–'80s and 2 larger series of 52 and 20 patients^10^, ^12^ collected by Internet recruitment and assessed by telephone interview.

BIID affects both sexes, with a male predominance.^10^, ^12^ Affected individuals describe a sense of paradoxical incompleteness of their body because of the presence of an undesired limb, most frequently a leg, that does not match their “inner self body image.”^10^, ^12^ This compulsive feeling usually starts in childhood and worsens progressively with age, causing overwhelming anxiety and suffering in most cases.^10^, ^12^ Pretending to be an amputee (bending their legs, using crutches or bandages) usually provides transient relief. Over time, most subjects seek an amputation, inflict injuries, or try to cut the limb themselves. The affected body part is always healthy and not painful. A sexual paraphilic component (sexual arousal on fantasizing about being an amputee or by the vision of an amputee) is reported in a minority of cases.^10^ Medical and psychological interventions are thought to be ineffective, although partial relief of the symptoms may be achieved.^13^

Very little evidence is available on the underlying mechanism behind BIID. A neurological model^14^–^16^ based on dysfunction in right hemisphere areas that mediate body representation has been proposed. In the presence of normal sensory afferent input, a mismatch between central representation and peripheral feedback is hypothesized to lead to conflict in perception of the affected limb. The evidence for this model is limited, however. Skin conductance responses below the level of the desired amputation are lower than above,^16^ and it has been suggested that this reflects a congenitally dysfunctional right superior parietal lobule (with resultant abnormal body image) that causes abnormal sympathetic outflow via the insula.^16^ This is in keeping with the results of an interview study of 20 patients with BIID, where a left-sided predominance of symptoms and descriptions of the affected limb that were similar to those of patients with somatoparaphrenia led the authors to conclude that nondominant frontal/parietal structures might be involved in the pathophysiology of the condition.^12^

Our fixed dystonia cases have physical signs and symptoms and in this regard are clearly different from people with apotemnophilia. However, cases with apotemnophilia demonstrate how a deficit in central body schema might be implicated in a desire for amputation, a phenomenon rarely encountered in patients with neurological signs outside the setting of fixed dystonia and CRPS (see below).

### People with Physical Signs and Pain Who Request Amputation

It is most uncommon for patients with disabling neurological diseases affecting the limbs in a focal or multifocal fashion, such as Parkinson's disease, stroke, multiple sclerosis, or neuropathy, to resort to amputation. In fact, there are only 3 case reports of this occurring in the medical literature, 1 due to a paralyzed limb after stroke,^17^ 1 due to painful contractured legs after spinal cord injury,^18^ and 1 in a child with secondary dystonia,^19^ However, we acknowledge that other cases may exist and have simply not been reported (and were alerted by an anonymous reviewer of this article that he/she had experience of 2 cases of amputation for osteomyelitis associated with abnormal postures due to corticobasal degeneration and Rassmussen's-like encephalopathy).

At first glance, therefore, the 5 cases with fixed dystonia who sought amputation appear highly unusual in terms of previously reported cases of amputation in neurological disease. However, there are several reports of amputation (combined producing a total of more than 50 patients), mainly in the orthopedic literature, in patients with CRPS1.^20^–^22^ There is considerable overlap between fixed dystonia and CRPS1 and evidence that at least a proportion of patients reported in these case series had fixed dystonia. Nine patients in 1 series^21^ asked for amputation because of “ankylosed, hypersensitive fingers,” and 4 in another series^22^ had “reduced range of motion” of the affected limb that led to the operation. Most cases were longstanding and refractory to alternative measures, and a handful underwent multiple amputations. The main indications were intractable pain, functional disability caused by the painful limb, and recurrent infections of the limb. The largest series reported the results of 34 amputations in patients with CPRS1.^21^ Nearly 40% had periprocedural complications (infection and delayed healing), phantom limb pain occurred in 85%, CRPS1 recurred in the stump in a similar number, and 2 developed CPRS1 in another limb. Only 2 cases had benefit in terms of pain reduction, and 9 had some degree of improvement in function. The poor outcome in these CRPS1 cases following amputation is mirrored by the 3 fixed dystonia patients in our series who achieved amputation of their limb. It was universally unsuccessful, and phantom limb pain and spread of symptoms was the only outcome.

It is possible that BIID, CRPS1, and fixed dystonia share a common abnormality in central body schema representation. In patients with BIID, this abnormality may be developmental and arises without a specific triggering event. In patients with CRPS1 and fixed dystonia, a peripheral painful stimulus might act as a trigger to destabilize central body schema. This is supported in fixed dystonia by our recent data^6^ and in “pure” CRPS1, where mental rotation abnormalities that correlate with duration of symptoms and perceived intensity of pain are found.^23^ With regard to dystonia, mental rotation abnormalities have also been reported in *DYT1* mutation carriers (both manifesting and nonmanifesting^24^) and primary hand dystonia,^8^ and therefore this abnormality is not specific to fixed dystonia. However, the coexistence of severe pain in patients with fixed dystonia may trigger or modulate body schema abnormalities in a different manner to primary dystonia, and in some patients this could lead to seeking amputation. One hypothesis is the interaction of a number of factors with a preexisting (developmental) abnormality in central body schema (as suggested in BIID). These factors include an underlying psychopathology that causes abnormal attentional focus on a painful injury, thereby increasing central plastic changes that occur normally after pain, voluntary or therapeutic immobilization of the limb (by patient or treating doctors, respectively), reducing normal afferent feedback from the limb leading to an abnormal increase in gain of excitability in central sensory areas. Many patients with stroke, neuropathy, or multiple sclerosis have painful and postured limbs, but do not seek amputation. In patients with fixed dystonia and those with CRPS1, the disorder appears to go beyond pain and posture to a deeper abnormal relationship with the limb, which in some leads to seeking amputation. This abnormal relationship might also extend to pursuit of other surgical interventions (cf, case 1), which are common in fixed dystonia. It would be possible to test these hypotheses, for example in functional imaging studies looking at response to painful stimuli with particular focus on nondominant parietal lobe structures and neurophysiological assessments of cortical excitability changes occurring with attention toward or away from the affected limb.

Amputations in CRPS1 and fixed dystonia are almost uniformly unsuccessful, as demonstrated by the medical literature and in our cases where amputation was achieved. Symptoms typically return in the stump or another body part, further supporting the notion of a disturbed central body schema. In view of this, we would strongly counsel physicians and surgeons against recommending or performing amputations in patients with CRPS1 and/or fixed dystonia, as the current weight of evidence is that such procedures are likely to do harm and are therefore unethical.

Fixed dystonia is a severely disabling condition affecting young adults. The theory outlined above generates some testable hypotheses regarding the pathophysiology of fixed dystonia that attempt to move beyond the dualistic battle between classifying these patients as organic or psychogenic toward a more integrated view of brain dysfunction in this enigmatic disorder.
